# Dwarfism in *Pinus taeda* originates from independent somatic mutations co-localized in a shared genomic region

**DOI:** 10.1038/s41437-025-00814-5

**Published:** 2025-12-12

**Authors:** Pinar Guner, M. Nasir Shalizi, Fikret Isik, Trevor D. Walker

**Affiliations:** Cooperative Tree Improvement Program, Department of Forestry and Environmental Resources, North Carolina State University, Raleigh, NC USA

**Keywords:** Mutation, Genetic markers, Plant genetics

## Abstract

Somatic mutations in long-lived conifers are rarely characterized yet offer a unique window into the spontaneous genetic forces that shape variation in plants. In *Pinus taeda*, dwarf phenotypes originate from abnormal branches, colloquially known as “witches’ brooms”, where progeny derived from the affected branch segregate for dwarfism in an apparent Mendelian 1:1 ratio. In this study, we genotyped six unrelated wind-pollinated families segregating for dwarfism using single-nucleotide polymorphism markers that had been previously positioned on a linkage map. Trait-loci association analyses identified a genomic region on linkage group eight (spanning 98-155 cM) that was strongly associated with dwarfism across unrelated families. This finding suggests that independent, de novo somatic mutations within a common genomic region are the basis for stable dwarf phenotypes in *P. taeda*. The implicated region is quite large and it remains to be determined if the same growth regulation gene or genes are responsible, but the shared region is evidence for disruption of a common pathway. To more formally describe the witches’ broom phenomenon and distinguish mutants from pathogen-induced brooms, we propose the Latin name *Ramus nanus mutatus*. We discuss the contribution of somatic mutations to variation in forest trees, the potential utility of the dwarfing mutation for rootstocks in forestry seed orchards, and the next steps toward characterizing the pathways underlying dwarfism and their homology in other conifer species.

## Introduction

Dwarfism in plants is a condition where an individual exhibits reduced overall size, stature, or growth compared to typical individuals within the species. This trait can be valuable in agriculture for benefits such as increased lodging resistance, increased fruiting, and facilitating fruit harvest (Sasaki et al. 2002; Hedden 2003; Elias et al. 2012). In some tree species, dwarf trees are prized by gardeners (Lütken et al. 2012). Dwarf phenotypes may result from genetic mutations, hormonal imbalances, environmental stresses, or specialized cultivation practices. Extensive studies in fruit tree species such as apple, peach, and pear have identified loci controlling dwarfism, predominantly involving hormonal regulation pathways (Knäbel et al. 2015; Hollender et al. 2016; Moritani et al. 2025). Gibberellins, in particular, have been frequently associated with dwarfism due to their central roles in cell elongation and division (Santner et al. 2009; Depuydt and Hardtke 2011). Other hormones, such as auxin and abscisic acid, have been implicated in fruit tree dwarfism (Jia et al. 2018; Liu et al. 2022). The genomic basis of dwarfism in conifers has not yet been well studied.

**Fig. 1:**
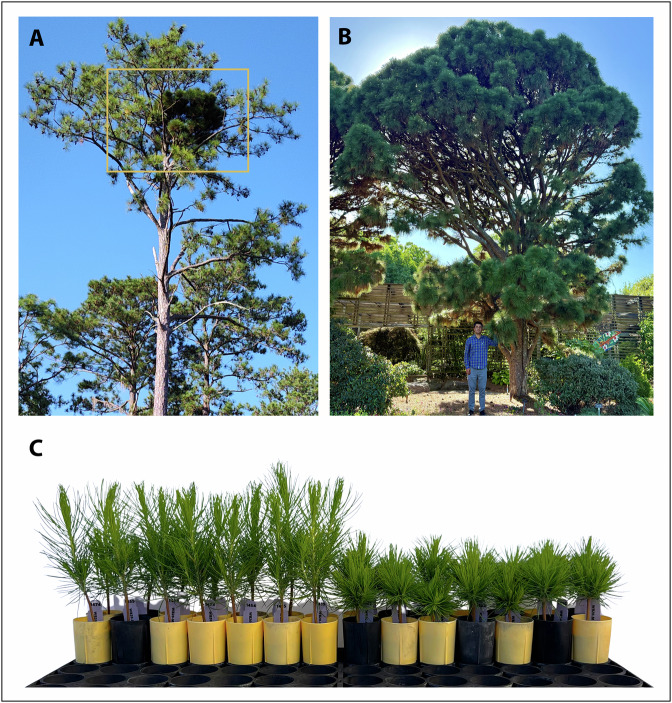
Representative dwarf phenotypes observed in *P. taeda.* **A** Witches’ broom on a mature tree in a natural setting. **B** Mature dwarf trees originated from a historically documented witches’ broom, grown at the J.C. Raulston Arboretum at NC State University. **C** Sixteen-week-old wind-pollinated seedlings collected from a witches’ broom (family WB-20), grown in a greenhouse, exhibited clear segregation for the dwarf phenotype. Photo credit: **A** Steve McKeand. **B**, **C** Authors.

In conifers, the dwarf phenotype most commonly originates as a witches’ broom (WB), which is an afflicted branch in an otherwise normal tree phenotype that is characterized by reduced shoot elongation, dense branching patterns, and decreased apical dominance (Yamburov et al. 2017; Polyakova et al. 2019) (Fig. 1). The term *witches’ broom* appears in European gardening chronicles as early as the 1850s, aptly describing the habit of the branch while also insinuating the branch has been “cursed” (Fordham 1967). Gardeners have long recognized that cones from WB, which tend to be much smaller than cones on adjacent normal branches, can produce offspring with dwarf phenotypes whereas adjacent normal cones do not, apparantly due to a heritable somatic mutation. In *Pinus taeda* (loblolly pine), such dwarf seedlings have been cultivated as compact, ornamental specimens in arboretums. Notably, in the 1960s, researchers at North Carolina State University collected seeds from a cone-bearing WB found on a *P. taeda* in Virginia, United States. Around half of these seeds produced dwarf seedlings, many of which were planted at the J.C. Raulston Arboretum in Raleigh, North Carolina, where they have grown into dense, short-statured trees with rounded crowns and given the cultivar name ‘Nana’ (Glover 1987) (Fig. 1).

Building on observations of *Picea abies* by von Tubeuf (1933) and *Pinus sylvestris* by Liess (1935), the heritable basis of dwarfism in WBs was first systematically demonstrated by Johnson et al. (1965) in *Pinus banksiana*. They showed that wind-pollinated seeds from WBs produced progeny segregating in a 1:1 ratio for dwarf and normal phenotypes whereas adjacent normal branches produced only normal progeny, demonstrating these WBs originated from somatic mutations. The same segregation for dwarfism was also observed in WB for multiple *Pinus* spp. by Fordham (1967) and for *Pinus sibirica* by Vasilyeva et al. (2020). The occasional appearance of WBs on sporadic trees across the landscape, without any pattern of outbreak or spread, further supported a genetic rather than pathogenic origin. Subsequent studies confirmed that while dwarf progeny maintained their reduced stature, their growth and developmental processes were otherwise comparable to their normal counterparts, including reaching reproductive maturity (Duffield and Wheat 1963). Furthermore, WB characteristics could be maintained through vegetative propagation in different environments (Fordham 1967; Waxman 1975). In contrast, pathogenic WBs caused by fungi (e.g., *Crinipellis perniciosa*, *Gymnosporangium* sp.), phytoplasmas, or viruses may result in temporary growth impairment and/or reduced reproductive capacity (Hogenhout et al. 2008; Bertaccini et al. 2014; Seo et al. 2017; Çağlar et al. 2023).

Despite the intriguing origins of dwarfism in *Pinus* spp. and its potential utility in horticulture or forestry (e.g., as rootstock to facilitate harvest of cones in seed orchards), its genomic basis remains largely uncharacterized. It remains unclear whether instances of dwarfism arising from unrelated and locally isolated WBs are caused by de novo mutations in the same genomic region or if multiple loci across the genome are responsible for this abnormal growth pattern. Considering that WB and their adjacent, unaffected branches originate from the same individual, they are genetically identical except for somatic mutations. This clonality provides a natural control for identifying the causal genetic changes underlying dwarfism. It would seem logical to sequence mutant and adjacent normal branches as a first approach to pinpoint the responsible mutation(s). However, identifying causal genes from non-targeted sequencing data would be analytically challenging for conifers with large, highly repetitive genomes in the absence of chromosome scale reference assemblies. For example, the most recent genome assembly for the 22 gigabase *P. taeda* genome remains highly fragmented at over 11 million contigs (Zimin et al. 2017). Another approach would be targeted sequencing of candidate genes, using a process of elimination to search for sequence variation between WB and adjacent normal branches. However, this strategy would be inefficient to determine the number of genes responsible, and the causal genes may not be included in the list of candidates.

A more tractable approach is trait-loci mapping, utilizing pre-designed single-nucleotide polymorphism (SNP) arrays or panels to genotype large progeny populations segregating for the trait. Trait-loci mapping, also known as genetic mapping or quantitative trait loci (QTL) mapping, relies on meiotic recombination among progeny to test for associations between SNP markers and phenotypes (Lynch and Walsh 1998). When marker order and genetic distance are known (i.e., through a linkage map), this approach can test hypotheses regarding the number and location of loci affecting the trait, and potentially identify SNPs in proximity to the causal gene(s). Trait-loci mapping has been successfully employed in fruit tree species to uncover loci associated with altered growth traits, including dwarfism and the ‘weeping’ growth habit, which can result from disrupted or reduced shoot gravitropism (Fresnedo-Ramírez et al. 2016; Hu et al. 2021; Kohler et al. 2023). In *P. taeda*, trait-loci mapping analyses have successfully uncovered genomic regions associated with disease resistance (Quesada et al. 2010; Lauer and Isik 2021). With the recent development of new SNP arrays/panels for *P. taeda* (Caballero et al. 2021; Lin et al. 2023) and a much-improved genetic map (Lauer and Isik 2021), there is a timely opportunity to investigate the genomic determinants of dwarfism originating from WB.

In this study, we collected wind-pollinated seed from seven cone-bearing WBs discovered in unrelated *P. taeda* trees, as well as from three Nana dwarf cultivars at the J.C. Raulston Arboretum, which are progeny from a WB. Wind-pollinated seedlings from families that segregated for dwarfism were genotyped using recently developed SNP marker arrays/panels (Caballero et al. 2021; Lin et al. 2023). Using a previously developed linkage map (Lauer and Isik 2021), trait-loci mapping techniques and genome-wide association study (GWAS) methods were employed to identify genomic regions associated with dwarfism.

The objectives of this study were to: (i) study the heritable nature of dwarfism in progeny derived from WB cones and dwarf trees, (ii) identify genomic regions associated with the dwarf phenotype, and (iii) determine whether the mutations underlying dwarfism in unrelated trees occur within the same genomic region. These results should enhance our understanding of the genetic basis of dwarfism in *P. taeda*.

## Materials and methods

### Plant population

Wind-pollinated cones were harvested from seven witches’ broom (WB) branches from unrelated and geographically dispersed *P. taeda* trees located throughout the Research Triangle region of North Carolina, USA, in October 2023. Trees with WB were identified from citizen science requests that were publicized through several local news outlets^1^. Out of more than 40 WBs reported, seven were identified for sampling due to the presence of cones and ease of access (Supplementary Table S1, Supplementary Fig. S1). The identification codes for the seven sampled branches were WB-04, WB-06, WB-07, WB-10, WB-20, WB-22, and WB-30. The sampled WBs occurred at heights ranging from around 4 to 25 meters above the ground. The age of sampled trees ranged from around 40 to 115 years old, determined using an increment borer. Cones from adjacent branches displaying normal phenotypes were also collected, and it was confirmed that their seeds did not segregate for dwarfism.

Wind-pollinated cones were also collected from three dwarf trees included in the Nana cultivar collection at the J.C. Raulston Arboretum at NC State University in Raleigh, NC. These three trees originated from wind-pollinated seed harvested from a single WB in Virginia, documented by Fordham (1967). The three Nana trees were confirmed as half-siblings following genotyping. Their identification codes were Nana-01, Nana-05, and Nana-10.

Seeds were sown under uniform nursery conditions in May 2024 at the Horticulture Field Laboratory at North Carolina State University, Raleigh, NC. Phenotypic evaluations began shortly after germination and were conducted regularly throughout the growing season. Final assessments were completed in November 2024. Measured traits included plant height, root collar diameter, and the incidence (yes = 1, no = 0) of the dwarf phenotype. A seedling was classified as dwarf if it was markedly shorter in height and exhibited a compact branching habit compared to its normal siblings (Fig. 1). In total, 1088 seedlings from seven WB families and three Nana families were assessed. Family size ranged from 67 to 134 offspring from the WB-derived cones, and 72 to 136 offspring from the Nana trees.

### DNA extraction

Leaf tissue was collected from individual seedlings 20 weeks after germination. Tissue was immediately transferred into individually labeled coin envelopes and stored in containers with silica desiccant beads to dry. Approximately 50 mg of dried leaf tissue per seedling was chopped into 1 mm segments to prepare for DNA extraction. Genomic DNA isolation was performed using a cetyltrimethylammonium bromide protocol specifically optimized for pine species by Cato and Richardson (1996), incorporating modifications based on Doyle and Doyle (1987) to ensure high-yield, high-quality DNA suitable for downstream genotyping. The DNA was extracted by the Forest Genomics Laboratory at the University of Florida.

### Genotyping

Genotyping was performed using two platforms specifically developed for *P. taeda*: the medium-density Pita50K Affymetrix SNP array (Caballero et al. 2021) and the cost-efficient, low-density AgriSeq targeted genotyping-by-sequencing platform (Lin et al. 2023). The latter platform was included to add study trees without exacerbating costs.

The Pita50K array comprises 46,439 high-confidence SNP and indel markers, curated by variant calling across compiled sequence datasets from trees spanning the species’ natural range, then empirically screened in a diverse panel to retain highly polymorphic markers (Caballero et al. 2021). The array was used to genotype maternal parents as well as seedling progeny from the Nana, WB-10, and WB-20 families. Genotyping with this array was conducted at the North American Genomics Laboratory through Applied Biosystems™ Axiom™ Genotyping Services (Thermo Fisher Scientific).

The AgriSeq platform was used to genotype seedling progeny from WB-07, WB-22, and WB-30 families. The AgriSeq marker panel was derived from 1018 high-quality SNPs selected from the Pita50K array and positioned on a linkage map developed by Lauer and Isik (2021). These markers are evenly distributed across the genome, with an average spacing of 3.09 cM and a maximum gap of 14.28 cM (see Lin et al. 2023, for details). All markers were mapped to the *P. taeda* reference genome (Pita v2.01; Zimin et al. 2017) and validated in silico for specificity and sensitivity by the AgriBusiness Bioinformatics Team in Austin, TX. The AgriSeq platform utilizes a highly multiplexed PCR-based approach to amplify targeted genomic regions in a single sequencing run (Carrasco et al. 2018).

The number of individuals genotyped per family and the number of markers retained after quality filtering are summarized in Table 1. The SNPs were filtered based on call rates ≥0.95 and minor allele frequencies ≥0.01. Only markers with positions on the previously developed linkage map (Lauer and Isik 2021) were included in the analyses.

**Table 1:** Mean height (cm) of dwarf and normal seedlings, incidence of dwarfism (%), number of seedling progeny per family, genotyping platform, and marker counts for 10 wind-pollinated *P. taeda* families collected from witches’ brooms (WB) and Nana trees.

Maternal Parent	Height (cm) ± SE		Dwarfism (%)	Genotyping Platform	Sample size (n)	Marker Count
	Dwarf	Normal				
WB-04	N/A	22.2 ± 0.27	0.0	N/A	67	N/A
WB-06	N/A	20.2 ± 0.29	0.0	N/A	134	N/A
WB-07	7.3 ± 0.12	15.4 ± 0.27	52.1	AgriSeq	103	1139
WB-22	8.4 ± 0.24	20.5 ± 0.43	24.2	AgriSeq	127	1229
WB-30	11.1 ± 0.22	20.6 ± 0.42	46.3	AgriSeq	95	1194
WB-10	8.7 ± 0.22	18.5 ± 0.41	53.8	Pita50K	120	7775
WB-20	9.9 ± 0.21	19.9 ± 0.50	49.6	Pita50K	119	7786
Nana*-*01	11.1 ± 0.28	19.2 ± 0.50	50.0	Pita50K	72	4577
Nana*-*05	9.4 ± 0.17	15.4 ± 0.24	55.8	Pita50K	136	6164
Nana*-*10	11.4 ± 0.22	20.5 ± 0.41	55.1	Pita50K	115	6729

The Nana-01, Nana-05, and Nana-10 parents originated from the same maternal WB and are half-sibs.

### Statistical analyses

#### Segregation and population structure

To evaluate whether dwarfism followed simple Mendelian inheritance patterns, a chi-square test was performed separately for each family to test for deviations of the observed number of dwarf and normal seedlings from the expected 1:1 segregation ratio.

To evaluate relatedness among individuals and characterize population structure, a genomic relationship matrix was computed from SNP marker data common in both the Pita50K and AgriSeq datasets using VanRaden’s method (2008):

1 $${\boldsymbol{G}}=\frac{\left({\boldsymbol{M}}-{\boldsymbol{P}}\right){\left({\boldsymbol{M}}-{\boldsymbol{P}}\right)}^{{\prime} }}{2\sum {p}_{i}\left(1-{p}_{i}\right)}$$

where ${\boldsymbol{M}}$ is the $n\,x\,m$ matrix of $n$ = 887 genotyped trees and $m$ = 400 markers containing the count of minor alleles (0, 1, or 2) for each genotype call, and ${\boldsymbol{P}}$ is the ${n\; x\; m}$ matrix containing twice the minor allele frequency at each marker locus $i$ ($2{p}_{i}$). The resulting ${\boldsymbol{G}}$ matrix is $n\times n$ with rows and columns corresponding to individual trees. Diagonal elements reflect inbreeding coefficients, and off-diagonal elements represent pairwise genomic relatedness based on marker sharing weighted by allele frequencies.

To visualize population structure, a principal components analysis was performed using centered gene content matrices, with the analysis performed separately by genotyping platform (Pita50K and AgriSeq) to retain more markers (9127 and 400 markers, respectively). The first two principal components were plotted to identify clustering patterns and to illustrate that the dwarf phenotype was not confounded with genetic background.

### Trait-loci mapping

To investigate regions of the genome associated with the dwarf phenotype, family-specific trait-loci mapping analyses were performed using the R/qtl package (v1.70) (Broman and Sen 2009). The phenotype was treated as binary (normal = 0, dwarf = 1), and genome-wide scans were conducted using the ${scanone}$ function with the expectation-maximization algorithm designed for binary traits.

Missing genotypes were imputed using the ${calc}.{genoprob}$ function under a hidden Markov model. Fine-scale jittering (${jittermap}$) was applied to avoid overlapping marker positions. Significance thresholds for QTL detection were determined using 1000 permutations ($\alpha$ = 0.05), with thresholds calculated separately for each family to accommodate differences in marker density and sample size. The QTL intervals were refined by estimating 95% Bayesian credible intervals with the ${bayesint}$ function of the R/qtl package.

Multi-locus modeling was conducted using ${makeqtl}$ and ${fitqtl}$ to assess the combined effects of multiple loci. In families with more than one peak exceeding the LOD threshold, multiple loci were initially modeled, and fine-mapping was performed using the ${refineqtl}$ function to optimize locus positioning and model fit. Epistatic interactions were tested using ${addint}$, but did not improve model fit and were excluded from the models.

The percentage of phenotypic variance explained (R^2^) by each significant marker was estimated using the ${fitqtl}$ function. Refined LOD profiles and QTL positions were visualized with plots generated in the ggplot2 package (Wickham 2016).

### Genome-wide association study

A genome-wide association study (GWAS) was conducted using seedlings from the Nana, WB-10, and WB-20 families, which were genotyped with the Pita50K SNP array. A total of 8259 SNP markers and 584 individuals were included in the analysis. Seedling height measured 20 weeks after germination was used as a continuous response to distinguish between dwarf and normal phenotypes. The GWAS was performed using the following linear mixed model:

2 $$y=\mu +{\alpha }_{i}{s}_{i}+{\boldsymbol{X}}b+{\boldsymbol{Q}}\delta +{\boldsymbol{Z}}a+e$$

where $y$ is the vector of the response variable (seedling height), $\mu$ is the overall mean, ${\alpha }_{i}$ denotes the additive effect of the ${i}^{{th}}$ SNP marker, and ${s}_{i}$ is the corresponding genotype call code as 0, 1, or 2. The term $b$ is the vector of fixed effects, which included the first five principal components derived from the genotype matrix (chosen based on a scree plot of eigenvalues) to correct for broad population structure and ${\boldsymbol{X}}$ is the corresponding design matrix. The vector $\delta$ captures additional population structure through eigenvectors derived from the marker matrix and ${\boldsymbol{Q}}$ is the corresponding design matrix. The $a$ is the vector of additive genetic effects scaled by the genomic relationship matrix ${\boldsymbol{G}}$, with expectations of $a\sim N\left(0,{\boldsymbol{G}}{\sigma }_{a}^{2}\right)$ and corresponding design matrix ${\boldsymbol{Z}}$. The $e$ is the vector of residuals with expectations of $e\sim N\left(0,I{\sigma }_{e}^{2}\right)$.

Prior to GWAS, markers were pruned for linkage disequilibrium using an R^2^ threshold of 0.80, and missing genotypes were imputed using population allele frequencies. These preprocessing steps were implemented using the ${ASRgenomics}$ package in R (Gezan et al. 2022). The GWAS was performed using the ${gwas}.{asreml}()$ function in the ${ASRgwas}$ pipeline. Genome-wide significance thresholds were determined using the Bonferroni correction at $\alpha =0.05$. Quantile-quantile plots were generated to assess the distribution of *P*-values under the null hypothesis and to evaluate potential confounding from population structure.

## Results

### Segregation of dwarfism

Among the seven field-collected WB families, five displayed clear segregation for dwarfism (Table 1). Based on chi-square tests, four families did not deviate significantly from the expected Mendelian 1:1 ratio (*P* > 0.05), with observed dwarfism rates ranging from 46% to 54%. For example, family WB-10 had 78 dwarf and 67 normal seedlings (χ^2^ = 0.83, *P* = 0.36), consistent with Mendelian segregation. One family (WB-22) showed a segregation pattern significantly different than 1:1, with only 24% dwarf seedlings (χ^2^ = 34, *P* < 0.01). The remaining two families (WB-04 and WB-06) did not produce any dwarf seedlings and were excluded from further genetic analyses. Their maternal witches’ brooms were likely of non-mutational origin, possibly caused by a pest or pathogen.

Dwarf seedlings averaged 40–60% (6.0–12.1 cm) shorter than their normal siblings at 20 weeks post-germination (Table 1). The height distributions of dwarf and normal phenotypes were distinct (Supplementary Fig. S2). Root collar diameter measurements were more similar between the two groups, and higher diameter-to-height ratios for dwarf seedlings reflected their compact habit.

### Population structure

**Fig. 2:**
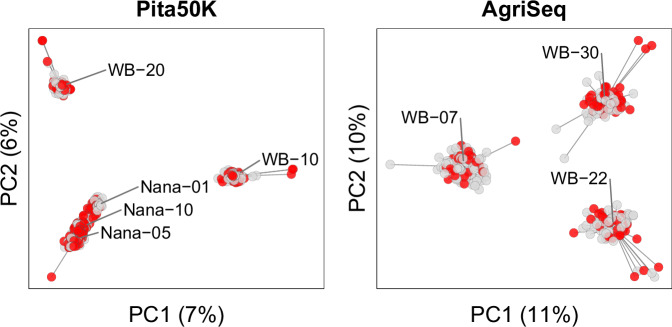
The first two principal components (PCs) of the genotype calls for 887 progeny from eight wind-pollinated families segregating for dwarfism (red circles are dwarf and gray circles are normal phenotypes). Families in the left plot were genotyped with the Pita50K Axiom™ array, and those in the right plot with the AgriSeq™ panel. The distinct clustering of families confirms the diversity of genetic backgrounds and illustrates that the dwarfism phenotype is not confounded with population structure.

Principal component analysis revealed clear genetic structure, with individuals clustering distinctly by family (Fig. 2, Supplementary Fig. S3). The Nana families formed a cohesive cluster, consistent with their shared grand-maternal origin. The WB families formed distinct clusters, reflecting their genetic independence. These patterns were supported by the genomic relationship matrix (Supplementary Fig. S4). Pairwise genomic relationship values within families were typically around 0.25 with occasional values near 0.50, suggesting a mix of half-sib and full-sib relationships, consistent with expectations from wind-pollination. Genomic relationship values among WB families were near zero, confirming their unrelatedness, while values among the three Nana families were closer to 0.125, reflecting their shared maternal grandparent. Both the principal components analysis and genomic relationship matrix demonstrate that the dwarf phenotype was not confounded with a particular genetic background.

### Trait-loci mapping

**Fig. 3:**
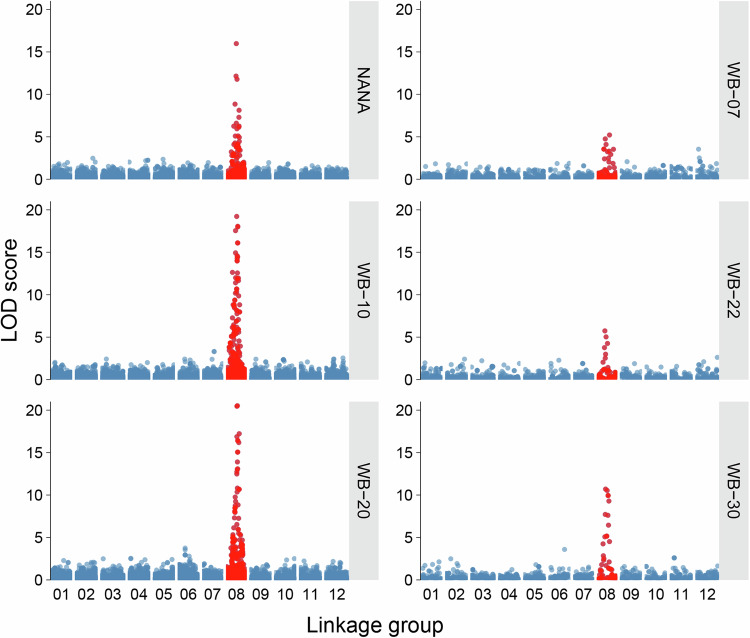
Associations between dwarfism incidence and marker genotypes from six independent QTL analyses. The three Nana families were combined for analysis. A common region on linkage group 8 was significantly associated with dwarfism in all families. The Pita50K array was used for Nana, WB-10, and WB-20, whereas the lower-density AgriSeq targeted GBS panel was used for WB-07, WB-22, and WB-30 families.

**Fig. 4:**
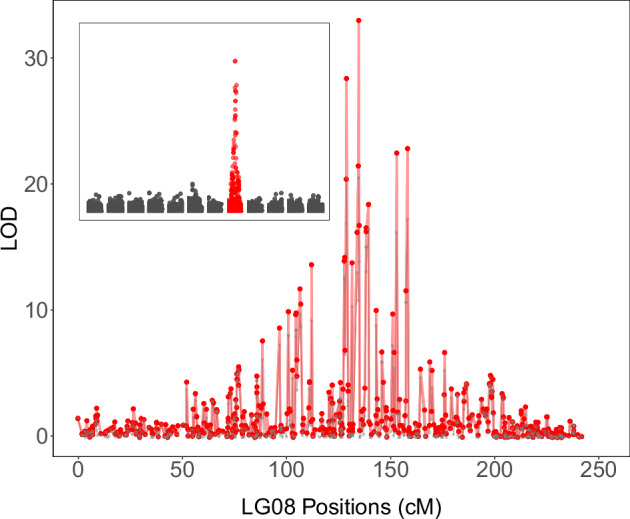
One-dimensional QTL scan for dwarfism in the WB-20 family of *P. taeda*, based on a binary trait model using the Expectation-Maximization algorithm. The main panel displays LOD scores across linkage group 8, where a prominent peak was detected at 134.2 cM. Refined QTL profiles (red) were derived by selecting significant markers, estimating Bayesian credible intervals, and re-optimizing locus position using the ${refineqtl}$ function in R/qtl. The inset panel summarizes genome-wide LOD scores across all 12 linkage groups, with linkage group 8 showing the only major association. The gray trace in the inset represents the original scan profile.

**Fig. 5:**
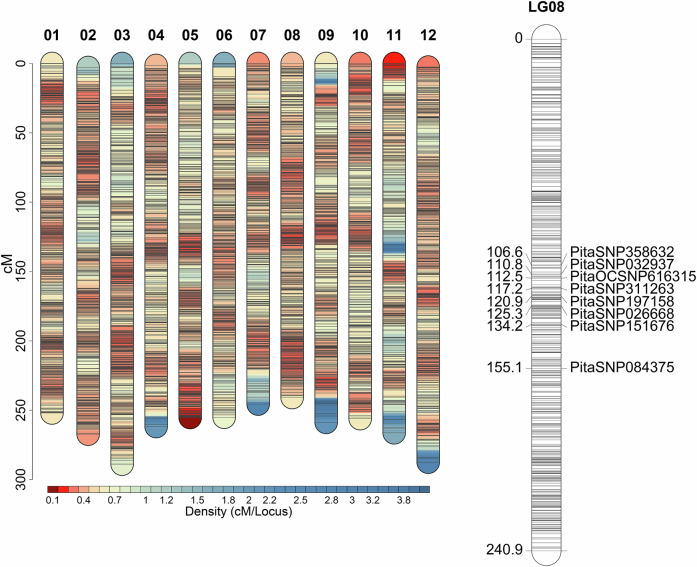
Linkage map of the 12 linkage groups, showing genetic positions in centimorgans (cM) along each group. Black lines represent individual marker positions. Marker density, expressed as cM per locus, is visualized with a heat scale. Warmer colors indicate higher marker density (lower cM/locus), while cooler colors indicate sparser regions. A detailed inset of linkage group 8 highlights the peak SNP markers associated with dwarfism.

Strong marker-trait associations with the dwarf phenotype were detected on linkage group 8 across all segregating families using independent trait-loci mapping analyses (Fig. 3). The most prominent signal was observed in the WB-20 family, where the maximum LOD score exceeded 20 and localized to the 128.4–134.2 cM interval on linkage group 8 (Fig. 4). This same genomic region showed significant associations in all families, and the peak markers within each family explained a substantial proportion of phenotypic variance (R^2^ values ranging from 17% to 56%) (Table 2). Across families, the most strongly associated markers clustered within a 20-30 cM window on linkage group 8 (Fig. 5).

**Table 2:** List of markers significantly associated with dwarfism in wind-pollinated families from five unrelated maternal witches’ brooms (WB) and three Nana dwarf trees.

Maternal Parent	Marker ID	LOD	Variance explained (%)	Position (cM)	Scaffold & Contig.	Ref Pos
WB-07	PitaSNP084375	5.21	21	155.1	S140341	38033
WB-22	PitaOCSNP616315	5.02	17	112.5	C4936665	15497
WB-30	PitaSNP358632	10.70	40	106.6	S55356	41973
WB-10	PitaSNP026668	19.22	53	125.3	S164596	57856
WB-20	PitaSNP151676	20.52	56	134.2	S229907	23832
Nana-01	PitaSNP311263	11.36	52	117.2	S155275	16141
Nana-05	PitaSNP197158	13.92	40	120.9	S91192	25438
Nana-10	PitaSNP032937	14.83	45	110.8	C4757651	8512

The LOD score, genetic position on linkage group 8 (cM), corresponding reference scaffold and contig, and reference genomic position (based on NCBI-BLAST) are provided using the genome assembly version Ptaeda2.0 (TreeGenes Database, 2017). Significant markers explained 17–56% of phenotypic variance.

### Genome-wide association study

**Fig. 6:**
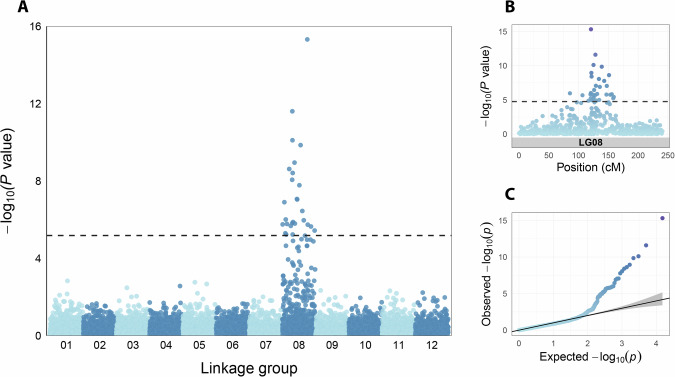
Genome-wide association analysis of seedling height across five wind-pollinated families segregating for dwarfism. **A** Manhattan plot showing degree of association for 8259 Pita50K SNP markers genotyped in 584 individuals, with a strong peak on linkage group 8. The dashed line represents the Bonferroni-corrected significance threshold (*P* < 0.05). **B** A close-up of the genomic region on linkage group 8, indicating significant associations between positions 85 and 160 cM. **C** Quantile-quantile plot illustrating that the majority of markers align with the null expectation (no association), while a small subset of SNPs, likely in linkage disequilibrium with the dwarfism QTL, display notable deviation, consistent with a true association signal.

To complement the linkage mapping analysis and validate genomic regions associated with dwarfism, GWAS was conducted using the Nana, WB-10, and WB-20 families (*n* = 584), while accounting for population structure and relatedness. A total of 8259 SNP markers were included in the analysis. The results reinforced a highly significant association peak on linkage group 8 (Fig. 6, Supplementary Fig. S5). The quantile-quantile plot showed that the observed *P*value distribution closely followed the null expectation for most markers, indicating effective control of population structure, and the notable upward deviation in the tail supported the presence of a true genetic association. A number of SNPs exceeded the genome-wide significance threshold based on a conservative Bonferroni correction ($\alpha$ = 0.05). The most significant marker-trait associations were concentrated within the 125-135 cM interval of linkage group 8 (Fig. 6), corroborating findings from independent family-level QTL scans.

**Fig. 7:**
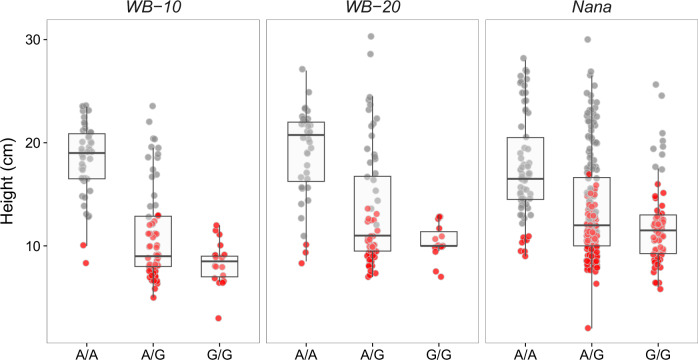
Height of seedlings segregating for dwarfism by genotype of the peak marker in the genome-wide association analysis. Dwarf and normal seedlings (red and gray points, respectively) are shown from two wind-pollinated witches' brooms (WB-10 and WB-20) and three Nana trees (combined). The marker (PitaSNP205637) segregated for all three genotype classes in these families.

The peak marker, PitaSNP205637, explained approximately 20% of the phenotypic variance and was segregating in the Nana families, WB-10, and WB-20 offspring. In WB-10 and WB-20, all offspring homozygous for the minor allele exhibited the dwarf phenotype (Fig. 7). In the Nana family, 55 of 64 individuals with the minor homozygous genotype displayed dwarfism. Approximately half of the heterozygotes at this locus exhibited dwarfism, while very few individuals homozygous for the major allele were dwarfs (14 out of 156). This genotype-phenotype pattern indicates proximity of the PitaSNP205637 marker to the causal region controlling dwarfism.

## Discussion

Dwarfism in *Pinus taeda* appears to originate from independent somatic mutations in a co-localized region of the genome. Using progeny or grand-progeny from six unrelated trees, each with an individual limb exhibiting the witches’ broom phenomena, we mapped de novo mutations causing heritable dwarfism to an interval on a single linkage group (chromosome). While dwarfism has been well-documented as segregating in progeny from mutant branches across various conifer species (Johnson et al. 1965; Fordham 1967; Vasilyeva et al. 2020), this study provides the first genomic mapping of the trait. Further characterization of this genomic region is necessary to determine if the responsible gene within each family is common across families, and assess the potential homology in a broader set of *Pinus* species and conifer taxa.

Not all trees with witches’ broom carry the heritable mutation for dwarfism. In this study, progeny from two of the seven sampled WB branches did not inherit the trait. These two branches likely developed dwarfism due to pathogenic or insect-related factors that disrupted normal growth processes. Such disturbances may alter hormone regulation, impair vascular tissue development, or interfere with cellular differentiation, leading to abnormal growth (Hogenhout et al. 2008; Bertaccini et al. 2014; Çağlar et al. 2023).

Historically, all dwarf branches have been labeled as “witches’ brooms,” a term that fails to distinguish between genetically stable mutations and pathogenic conditions. To clarify the distinction between genetic and pathogenic origins of dwarfism in individual tree limbs, and to provide a more formal alternative to the colloquial term “witches’ broom”, we propose the Latin term *Ramus nanus*, with suffix qualifiers *Ramus nanus mutatus* and *Ramus nanus pathogenicus* to differentiate the stable, heritable mutations from temporary, pathogenic witches’ brooms, respectively. This Latin terminology offers the international scientific community a more formal and precise description of the growth anomaly commonly named “witches’ broom”. With more complete characterization of the genes and/or pathways in this region, a refined assay could be developed for novel *Rami nani* to determine if their origin is mutational.

The genomic region associated with dwarfism was consistently mapped to linkage group 8 of the genetic map developed by Lauer and Isik (2021), specifically within the ~110-155 cM interval. We anticipated successful mapping within each family due to the distinct phenotype and simple inheritance pattern of the trait, and we were intrigued to find a shared genomic region across unrelated families. Notably, the locus was consistently identified across two genotyping platforms (the medium-density Pita50K SNP array and the lower-density AgriSeq targeted genotyping-by-sequencing panel) and two different statistical analyses (QTL mapping for the dwarf phenotype and GWAS for seedling height).

The wind-pollinated progeny population used in this study was not conducive to fine-mapping the locus causing dwarfism within each family, and the fragmented state of the reference genome precludes estimation of the physical size of the locus or investigating the presence of homologs within the interval. It remains to be determined if these independent mutation events are affecting a single major gene or a cluster of loci, or are the same type of mutation (e.g., substitution, insertion, deletion, repeat). The single, common association peak suggests disruption of an essential growth regulation pathway common across genetic backgrounds. Extensive research in crops and model plants has linked dwarfism to alterations in hormone biosynthesis or signaling, particularly involving gibberellins, auxins, and brassinosteroids. For instance, the ‘Green Revolution’ genes, such as *Rht* genes in wheat and *sd1* and *slr* genes in rice, encode proteins involved in gibberellin signaling (DELLA proteins) or biosynthesis, respectively, leading to reduced stem elongation (Sasaki et al. 2002; Hedden 2003). Mutations affecting auxin transport or response (e.g., affecting PIN-FORMED transporters or AUX/IAA repressors) or defects in brassinosteroids synthesis or perception (e.g., mutations in the BRI1 receptor) are well-documented causes of dwarfism across various plant species, including trees. Mutations affecting gibberellin biosynthesis and auxin transport can contribute to reduced growth and altered branching architecture in peach trees (Hollender et al. 2016). A dwarf apple mutant had defects in auxin response pathways involving Aux/IAA repressors, leading to impaired cell elongation (Jia et al. 2018). Mutations impacting abscisic acid signaling pathways have been implicated in growth defects and dwarfism in pear trees (Liu et al. 2022). While conifers have not been studied as extensively as fruit orchard angiosperms, the gibberellin-DELLA signaling module has been demonstrated to be evolutionarily conserved in *Pinus* spp. (Du et al. 2017). It is possible that hormone-related dwarfism mechanisms are conserved across woody perennials, and the major locus we discovered harbors a gene homologous to well-known pathways that affects cell division, elongation, or differentiation processes that determine plant stature. Further investigations, such as fine-mapping and the candidate gene approach, are needed to determine whether the locus or loci function through gibberellins, auxin, brassinosteroids, or other less-commonly implicated pathways such as abscisic acid or strigolactone signaling.

The inheritance of dwarfism segregated in an approximately 1:1 ratio of dwarf to normal progeny in all but one family, consistent with Mendelian expectations for a heterozygous dominant rare allele. Similar segregation ratios have been documented in other pine species (Johnson et al. 1965; Waxman 1975; Belokon et al. 2022), suggesting that dwarfism in *P. taeda* is governed by a single, dominant gene action. One exception in our dataset exhibited a lower-than-expected frequency of dwarf progeny (WB-22 with ~25% dwarfism), which may have been the result of reduced fitness factors such as diminished seed germination or impaired early seedling vigor in dwarf phenotypes. Further investigation of this *Ramus nanus mutatus* is warranted before concluding whether the deviation reflects a different inheritance pattern. While the observed segregation rates suggest dominance, creation of a homozygous dwarf individual is necessary to confirm this gene action. We would not expect the existence of a naturally occurring homozygous dwarf, which requires mating between two dwarfs, given the low selfing rates *of P. taeda* (Williams 2008) and the distance between geographically isolated *Rami nani mutati*. Controlled-pollination experiments are needed to clarify whether homozygous individuals exhibit a stable, exacerbated, or even nonviable phenotype.

Dwarf seedlings were consistently 30 - 50% shorter than their normal-stature siblings, while their girth tended to be more similar, resulting in a compact habit. The dwarf phenotype in the Nana cultivars, which have been maintained in the J.C. Raulston Arboretum for over five decades, demonstrates the stability of the phenotype (Fordham 1967). Interestingly, progeny from these first filial generation dwarfs also segregated for dwarfism and were indistinguishable from progeny of more recent *Rami nanus mutatus*. This intergenerational fidelity further supports that a stable heritable mutation confers dwarfism.

Because they are clonal, the *Rami nani mutati* and their adjacent unaffected branches present a unique opportunity for comparative genomics to identify and validate candidate genes underlying dwarfism. Transcriptomic analyses of apical meristems or elongating tissues from dwarf versus normal siblings may uncover downstream gene expression differences and help elucidate the disrupted molecular pathways. Although challenging due to the fragmented *P. taeda* reference genome, annotation and functional validation of candidate genes remain key objectives for future research.

### Contribution of somatic mutations to variation in trees

The extent to which somatic mutations contribute to heritable genetic variation in tree populations has not been thoroughly characterized. As a tree grows, new branches arise from the shoot apical meristem, a population of slowly dividing stem cells at the growing tip (Lyndon 1998). Mutations in these cells can become fixed within large portions of the plant that share cell lineages, creating an intraorganism genetic mosaic over time in a phenomenon termed ‘somatic genetic drift’ (Reusch et al. 2021). Although a small fraction of somatic mutations may improve fitness, most are expected to be neutral or mildly deleterious. Strongly deleterious mutations that compromise branch vigor are typically eliminated through intraorganismal selection, as shaded branches senesce and die. This is a likely fate for new *Rami nani* unless they occur in trees alongside roads or other clearings. Deleterious mutations that are recessive may persist in a heterozygous state, gradually accumulating in the genome and contributing to the population’s genetic load. The predominance of neutral mutations observed in forest trees, and their outcrossing reproductive biology to avoid inbreeding supports these expectations (Scofield and Schultz 2005; Eyre-Walker and Keightley 2007).

Somatic mutations are not limited to plants; all organisms inevitably acquire mutations through DNA replication errors or environmental stressors. However, animals differ fundamentally in their strategy to protect their hereditary lineage. Animals establish a dedicated germline early in development, long before sexual maturity, thereby insulating gametes from the mutational history of somatic tissues. Plants do not form a segregated germline per se, and there is some debate on the timing or even presence of plant germlines (Lanfear 2018). Some have argued that the shoot apical meristem acts as a functional germline, reducing the accumulation and transmission of somatic mutations through very slow division rates of central stem cells and other cell and tissue mechanisms (Burian 2021). Our findings provide a clear counterexample to, or breaches of, the plant germline. Although the mutation causing *Ramus nanus* may be rare, the abundance of *P. taeda* in the region and the conspicuous dwarfing phenotype give ample opportunity to observe the spontaneous genetic forces that shape variation in plants.

### Implications for forestry seed orchards

Known for its rapid growth and apical dominance, *P. taeda* is the predominant timber species in the southeastern United States and has undergrown several cycles of breeding for improved growth, stem straightness, and disease resistance (Isik and McKeand 2019). Genetic gains are delivered to forest landowners through seed produced in grafted clonal seed orchards (McKeand 2019). The species’ rapid growth and pronounced apical dominance create challenges in seed orchards, as breeding, seed harvesting, and orchard management require aerial lifts (i.e., bucket trucks or boom lifts). Operating this large, slow-moving equipment during the time-sensitive and fast-paced flowering season presents safety hazards, logistical complexities, and considerable expense. Dwarf rootstocks are successfully utilized in horticultural species such as apple, apricot, and peach to facilitate fruit harvest (Webster and Webster 2002). Over the past century, the development of apple rootstocks such as the Malling (e.g., M.9 and M.27) and Geneva series has transformed orchard systems by enabling higher-density planting, improving labor efficiency, and enhancing precocity (Marini and Fazio 2018). Parallel outcomes have been realized in other fruit trees, including the Controller series in peach and Gisela rootstocks in cherry (DeJong et al. 2005; Whiting and Lang 2004). These advances in orchard management culminated after decades of research into the physiological and genetic basis of dwarfing. Previous studies have attempted to identify dwarfing rootstocks for pine seed orchards (Jayawickrama et al. 1997), although these studies focused on rootstocks from slower-growing families with typical phenotypes rather than mutant dwarfs. Naturally occurring *Rami nani mutati* may present a promising genetic resource for dwarf rootstocks in pine seed orchards.

## Conclusions and future directions

This study revealed a clear Mendelian segregation pattern for dwarfism in *P. taeda*, even across two generations, and mapped a major gene or set of genes on underlying the trait across a diverse set of trees. These mutations originated somatically and independently in what are colloquially referred to as “witches’ brooms”, although some branches exhibiting this phenomenon are of pathogenic origin rather than mutation, and their progeny do not inherit the phenotype. We propose the Latin terms *Ramus nanus mutatus* and *Ramus nanus pathogenicus* to distinguish the two and provide a more formal terminology for the international research community. This mutation may find utility as dwarf rootstocks in seed orchards and enhance the deployment of genetic gain in pine plantations for timber production. To further characterize the function of the associated genomic region, the population described in this study is undergoing bulked-segregant analysis, targeted sequencing, applications of hormones in a greenhouse environment (to evaluate if the normal phenotype can be recovered), and controlled-pollination to produce full-siblings for fine-mapping.

## Supplementary information

Supplementary information

## Data Availability

The data and the code are archived on GitHub: https://github.com/pgnrnr/dwarfism.

## Footnotes

1 https://www.wunc.org/environment/2023-10-24/witchs-witch-brooms-loblolly-pine-trees-improvement-program-nc-state-university-seed-halloween-citizen-science https://cnr.ncsu.edu/news/2023/10/witchs-brooms-tree-improvement-program/ https://newbernlive.org/nc-forestry-is-looking-for-witch’s-broom-p4120-174.htm Last accessed June 1, 2025
