# Supplementary material for: Dwarfism in Pinus taeda originates from independent somatic mutations co-localized in a shared genomic region
Source: Heredity (Edinb). 2025 Dec 12;135(1):23–33. doi: 10.1038/s41437-025-00814-5 (PMC12811388; doi:10.1038/s41437-025-00814-5)

**Supplemental Tables and Figures for the manuscript: Dwarfism in *Pinus taeda* originates from independent somatic mutations co-localized in a shared genomic region**

Pinar Guner, M. Nasir Shalizi, Fikret Isik, Trevor D. Walker

Author affiliations: Cooperative Tree Improvement Program, Department of Forestry and Environmental Resources, North Carolina State University, Raleigh, NC 27695, USA

**Table S1.** Geographic locations and phenotypic characteristics of the *P. taeda* trees with witches’ brooms sampled across the Research Triangle Park region in North Carolina. Cones were collected in October 2023.

| tree id | Latitude | Longitude | Tree height (m) | Branch height (m) | DBH  (cm) | Tree age (yr) |
| --- | --- | --- | --- | --- | --- | --- |
| WB-04 | 35.769 | -78.691 | 25.4 | 3.7 | 63.0 | 45 |
| WB-06 | 35.782 | -78.678 | 23.4 | 7.6 | 59.9 | 64 |
| WB-07 | 35.789 | -78.807 | 33.8 | 24.7 | 46.5 | 115 |
| WB-10 | 35.807 | -79.016 | 21.9 | 7.6 | 103.4 | 100 |
| WB-20 | 35.667 | -78.920 | 32.0 | 21.3 | 74.9 | 100 |
| WB-22 | 35.658 | -78.792 | 20.0 | 8.2 | 60.5 | 40 |
| WB-30 | 35.616 | -78.621 | 20.9 | 3.6 | 73.2 | 53 |

**Fig. S1.** Photos of *Pinus taeda* trees with *Ramus nanus mutatus* sampled in this study. A professional tree climber was hired to collect needle and cone samples from taller trees.**
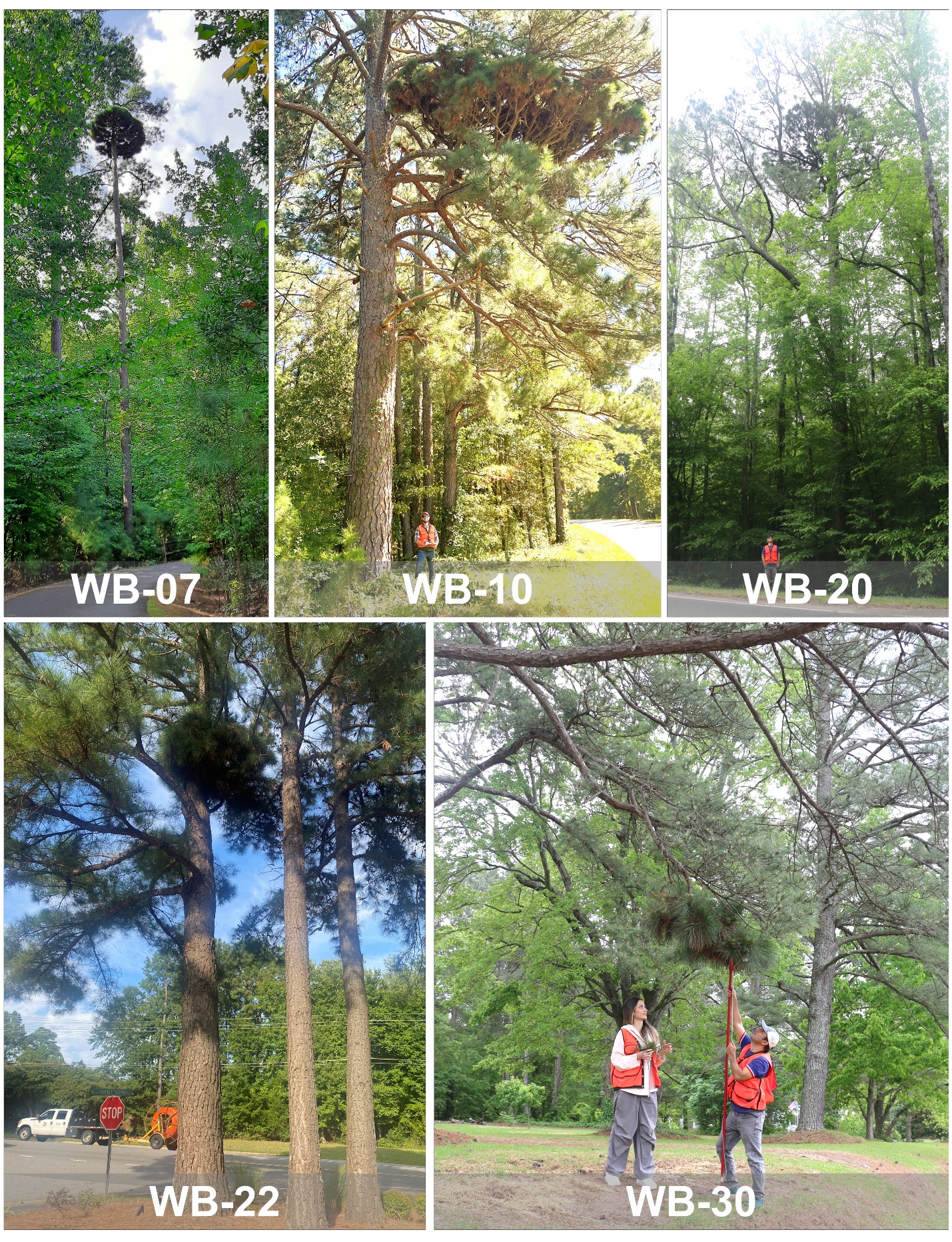
Fig. S2.** Distribution of height (left) and stem diameter-to-height ratio (right) for the dwarf (red) and normal (gray) individuals across eight *Pinus taeda* families. Dwarf seedlings were considerably shorter in height and were very compact (higher diameter-to-height ratio) compared to their normal siblings. These patterns were observed in progeny from both dwarf Nana trees from an arboretum and field-collected witches’ brooms.


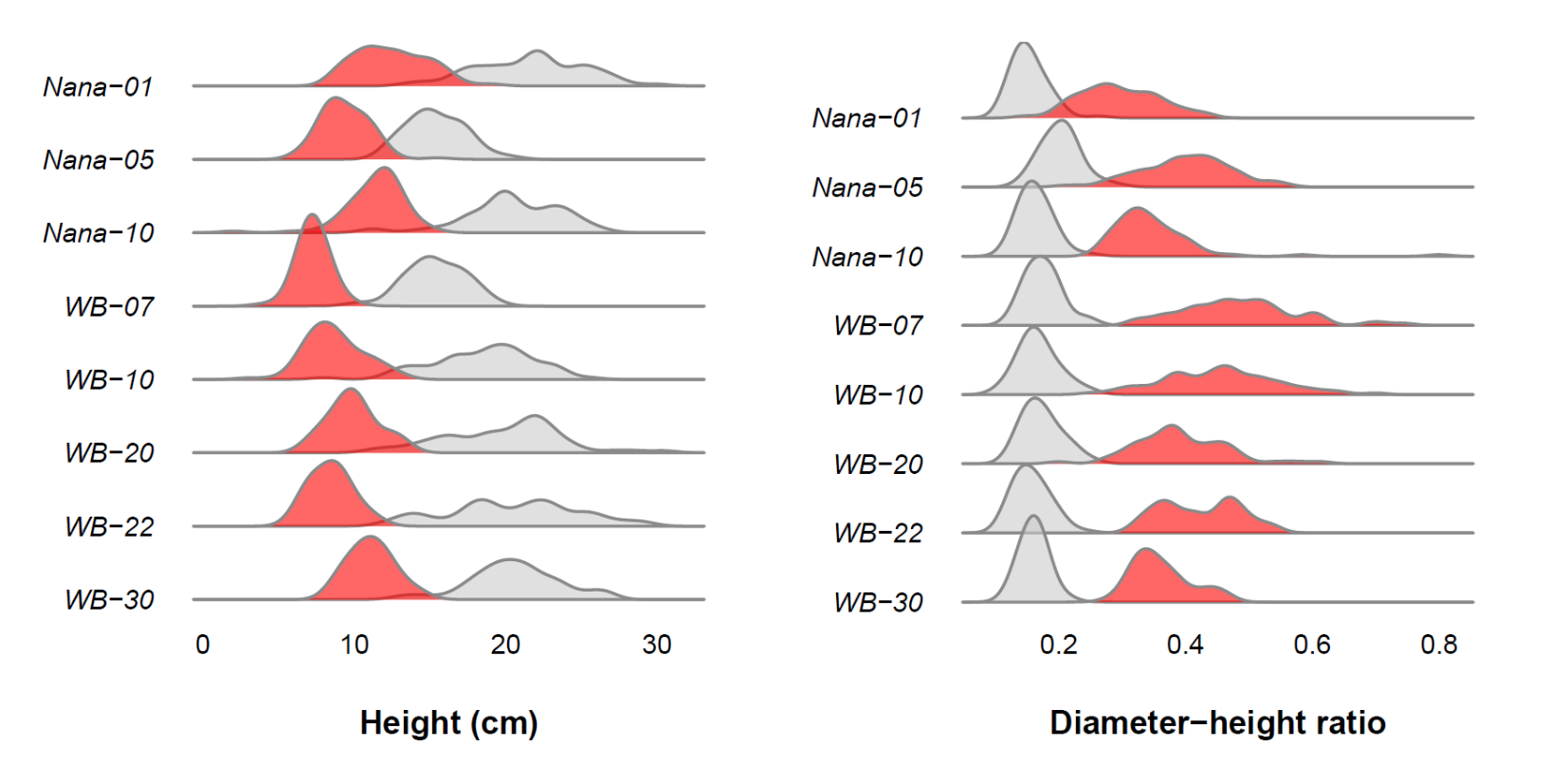


**Fig. S3.** The first two principal components (PCs) of the genotype calls for 887 progeny from eight wind-pollinated families segregating for dwarfism genotyped using a combined dataset with the Pita50K Axiom™ Array and the AgriSeq™ panel (500 SNP markers). The distinct clustering of families confirms the diversity of genetic backgrounds.


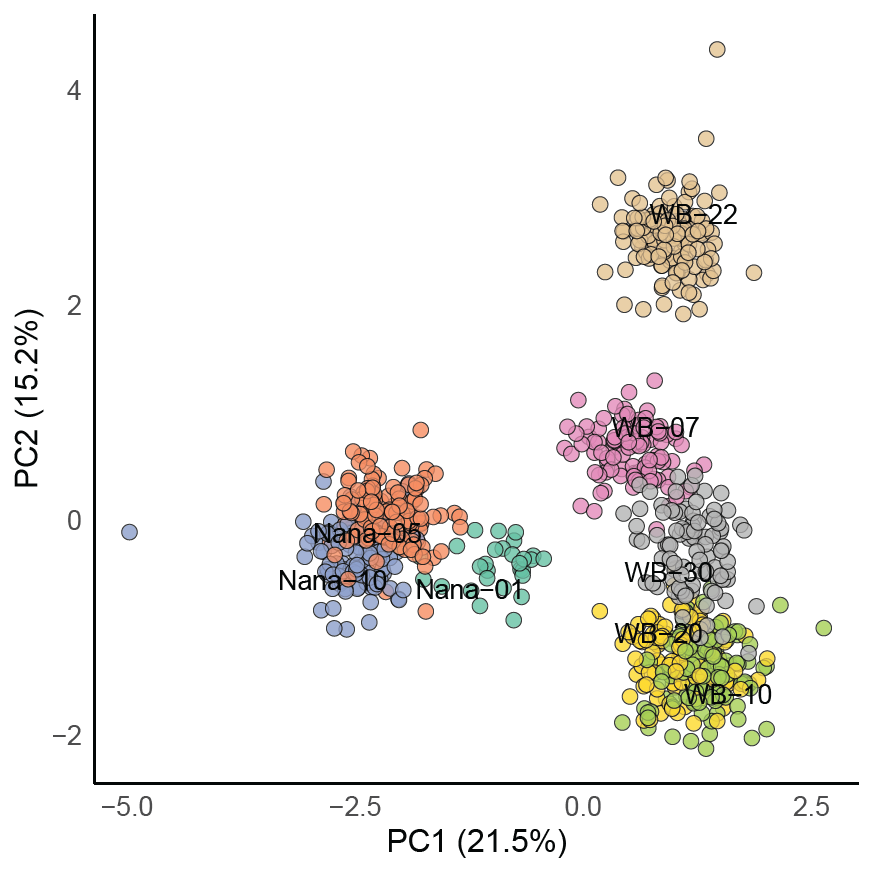


**Fig. S4.** Genomic relationship matrix among 887 individuals from eight wind-pollinated families. Each cell represents a pairwise genomic relationship coefficient between individuals, calculated using 500 SNP markers. Darker blue blocks along the diagonal reflect within-family relationships, with most values indicating half-sibling relationships and fewer indicating full-sibling relationships (particularly in WB-07 and WB-30). The three Nana families in the left lower corner share a common grandparent and have values reflecting first-degree cousin-level relatedness. The off-diagonal values between other families indicated no relatedness.
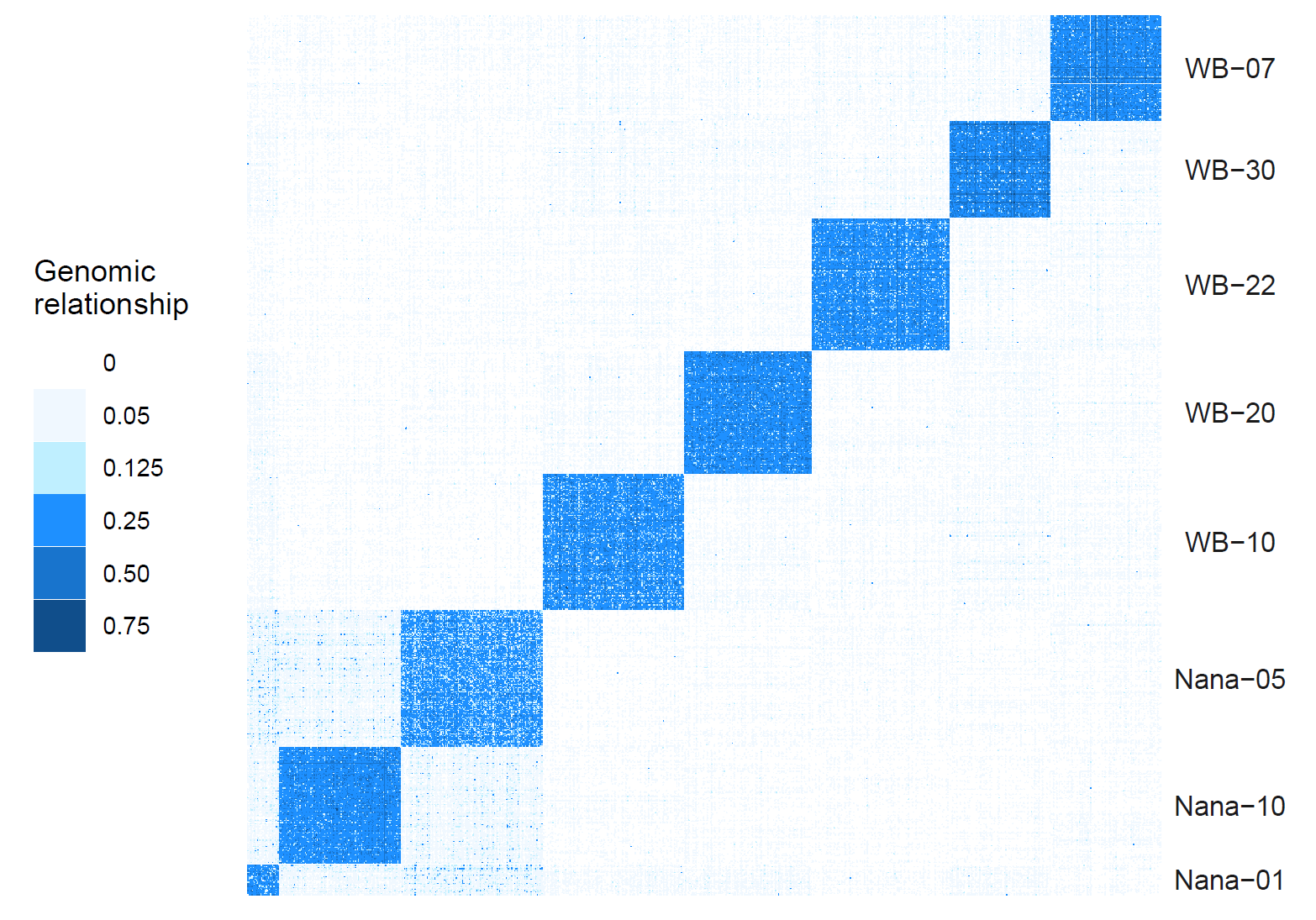


**Fig. S5.** Genome-wide association analysis of dwarfism (as a binary trait) across eight wind-pollinated families segregating for dwarfism, with Manhattan plot showing degree of association for 400 SNP markers genotyped in 839 individuals, with a strong peak on linkage group 8. The dashed line represents the Bonferroni-corrected significance threshold (P < 0.05).


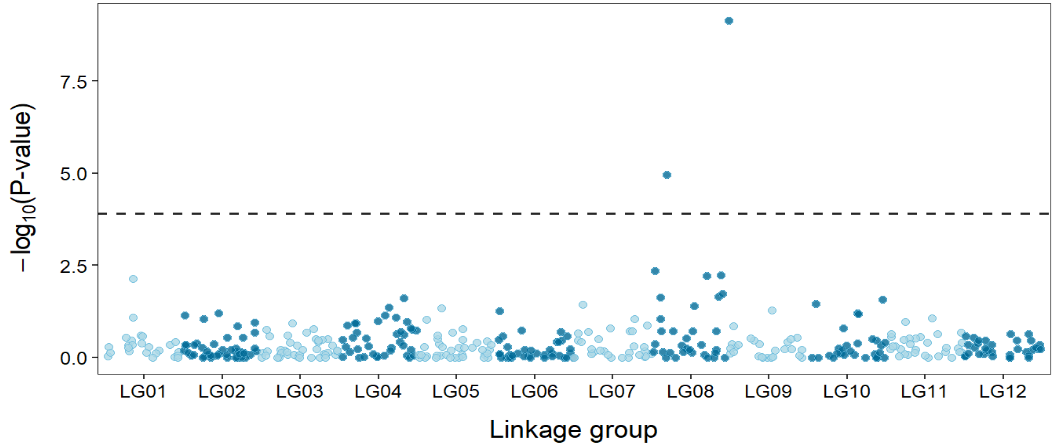

Supplement: Supplementary file 1 — Supplementary information [file 41437_2025_814_MOESM1_ESM.docx]
